# Supplementary material for: STARD3 mediates non-vesicular cholesterol transport in Caenorhabditis elegans
Source: J Lipid Res. 2026 Aug 5;67(9):101118. doi: 10.1016/j.jlr.2026.101118 (PMC13571226; doi:10.1016/j.jlr.2026.101118)
Supplement: Supplementary file 1 [file mmc1.pdf]

Supporting Information

STARD3 mediates non-vesicular cholesterol transport in *Caenorhabditis elegans*

Bernabe Battista[1], Agustina Sambrailo[1], Franco A. Biglione[1], Verónica A. Lombardo[1,2], María C. Mansilla[1,3], Daniela Albanesi[1,3,4], María-Natalia Lisa[1,4], Diego de Mendoza[1*] and Andres Binolfi[1,4*]

[1]Institute of Molecular and Cellular Biology of Rosario, National University of Rosario (IBR-CONICET-UNR), Ocampo y Esmeralda, 2000 Rosario, Argentina

[2]Center of Interdisciplinary Studies (CEI), National University of Rosario (UNR), Maipú 1065, 2000 Rosario, Argentina

[3]Deptartment of Microbiology, School of Biochemical and Pharmaceutical Sciences, National University of Rosario (UNR), Suipacha 531, 2000 Rosario, Argentina

[4]Argentinian Platform of Structural Biology and Metabolomics (PLABEM), Ocampo y Esmeralda, 2000 Rosario, Argentina

***To whom correspondence should be addressed:**

Dr. Diego de Mendoza: Institute of Molecular and Cellular Biology of Rosario (IBR-CONICET-UNR). Postal address: Ocampo y Esmeralda, 2000, Rosario, Argentina. Phone: +543414237070 Int. 634/E-mail address demendoza@ibr-conicet.gov.ar

Dr. Andres Binolfi: Institute of Molecular and Cellular Biology of Rosario (IBR-CONICET-UNR). Postal address: Ocampo y Esmeralda, 2000, Rosario, Argentina. Phone: +543414237070 Int. 649/E-mail address binolfi@ibr-conicet.gov.ar

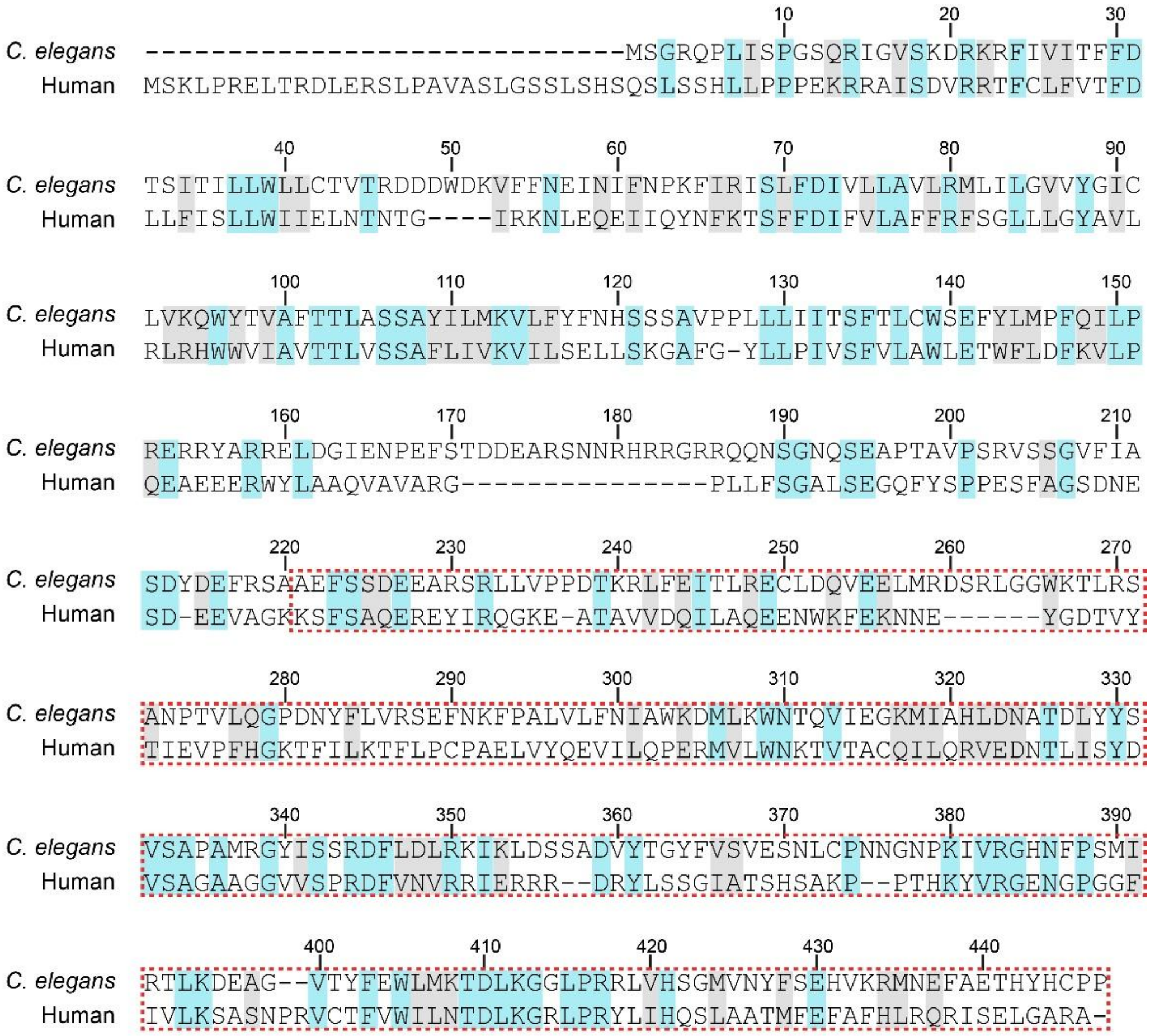

Supplementary Figure 1. Primary sequence alignments between *C. elegans* STARD3 (top, UniProt Q19819) and human STARD3 (bottom, UniProt Q14849). Identical and similar residues are shaded in cyan and gray, respectively. Red dashed boxes indicate the amino acid sequence of *C. elegans* START domain used in this work. The purified sequence contains an extra G residue at the N-terminus from the TEV cleavage site.

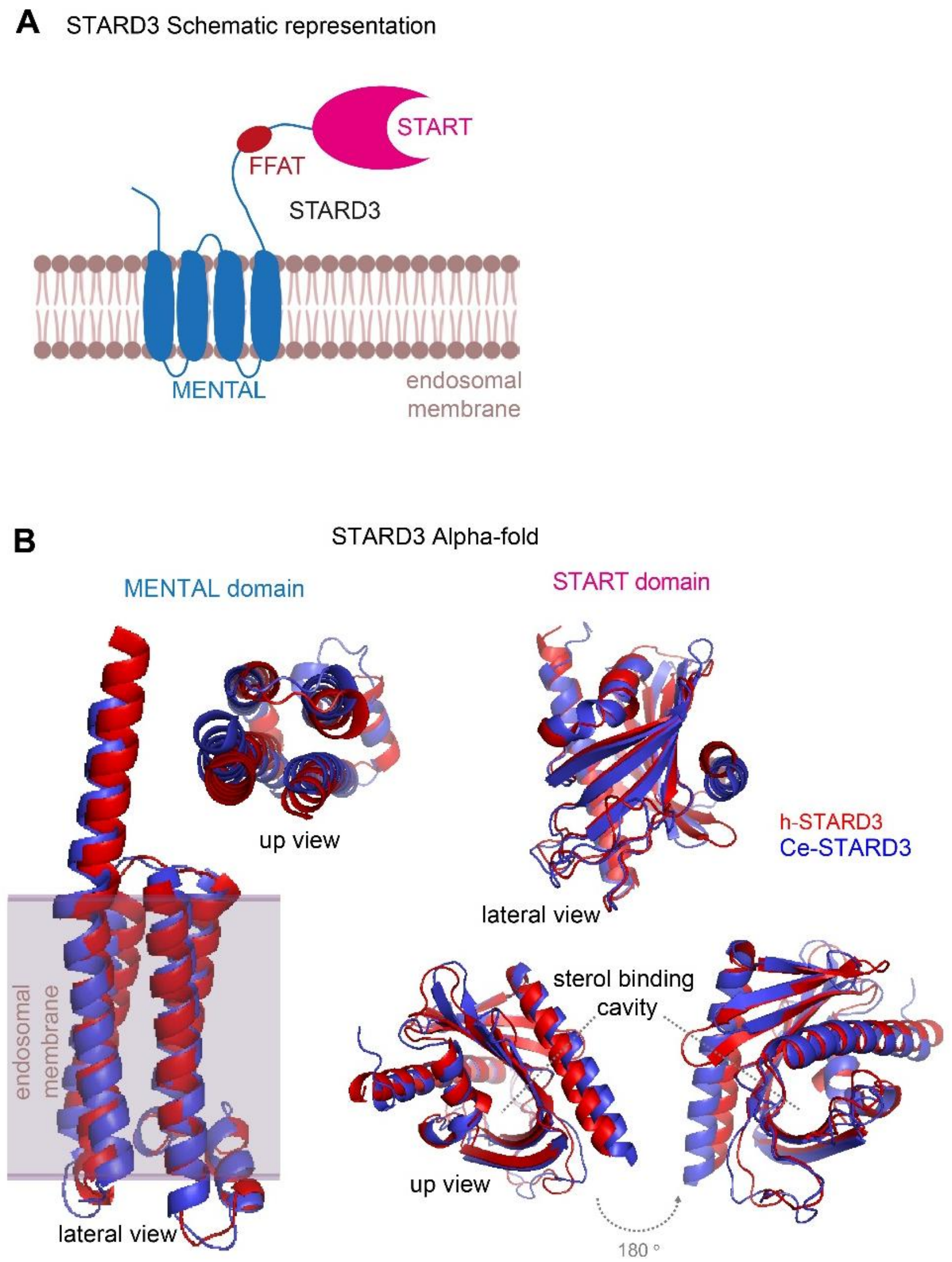

Supplementary Figure 2. (A) Schematic representation of STARD3 protein. The START, FFAT and MENTAL domains are depicted in pink, red and cyan, respectively. **(B)** Overlay of the AlphaFold-predicted 3D structures of human STARD3 (red) and Ce-STARD3 (blue). MENTAL domain is illustrated within the endosomal membrane.

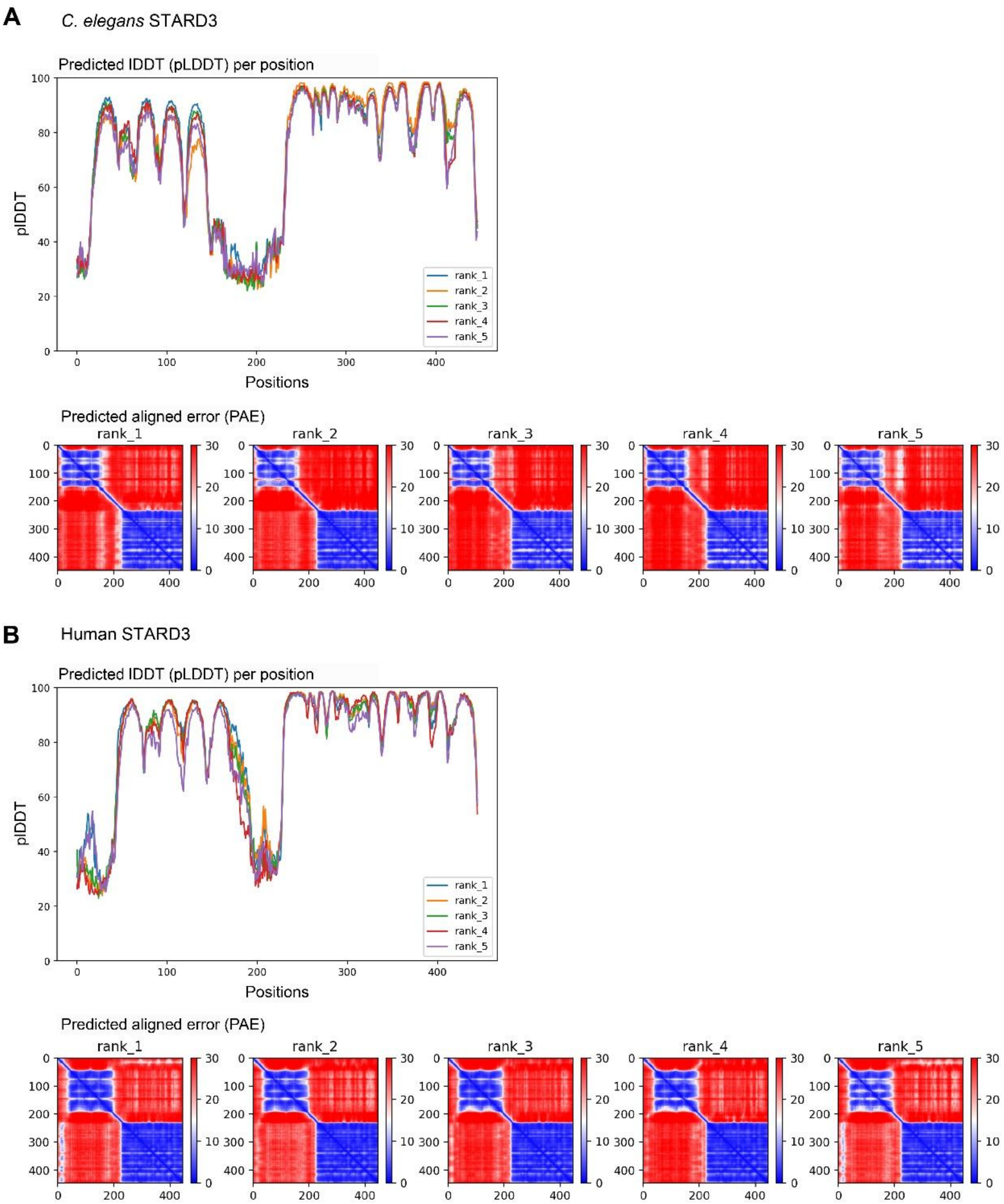

Supplementary Figure 3. Predicted local distance difference test (pLDDT) scores (top) and predicted aligned error (PAE) (bottom) for the AlphaFold2 models of *C. elegans* (**A**) and human (**B**) STARD3 proteins shown in **Supplementary Fig. 2b**.

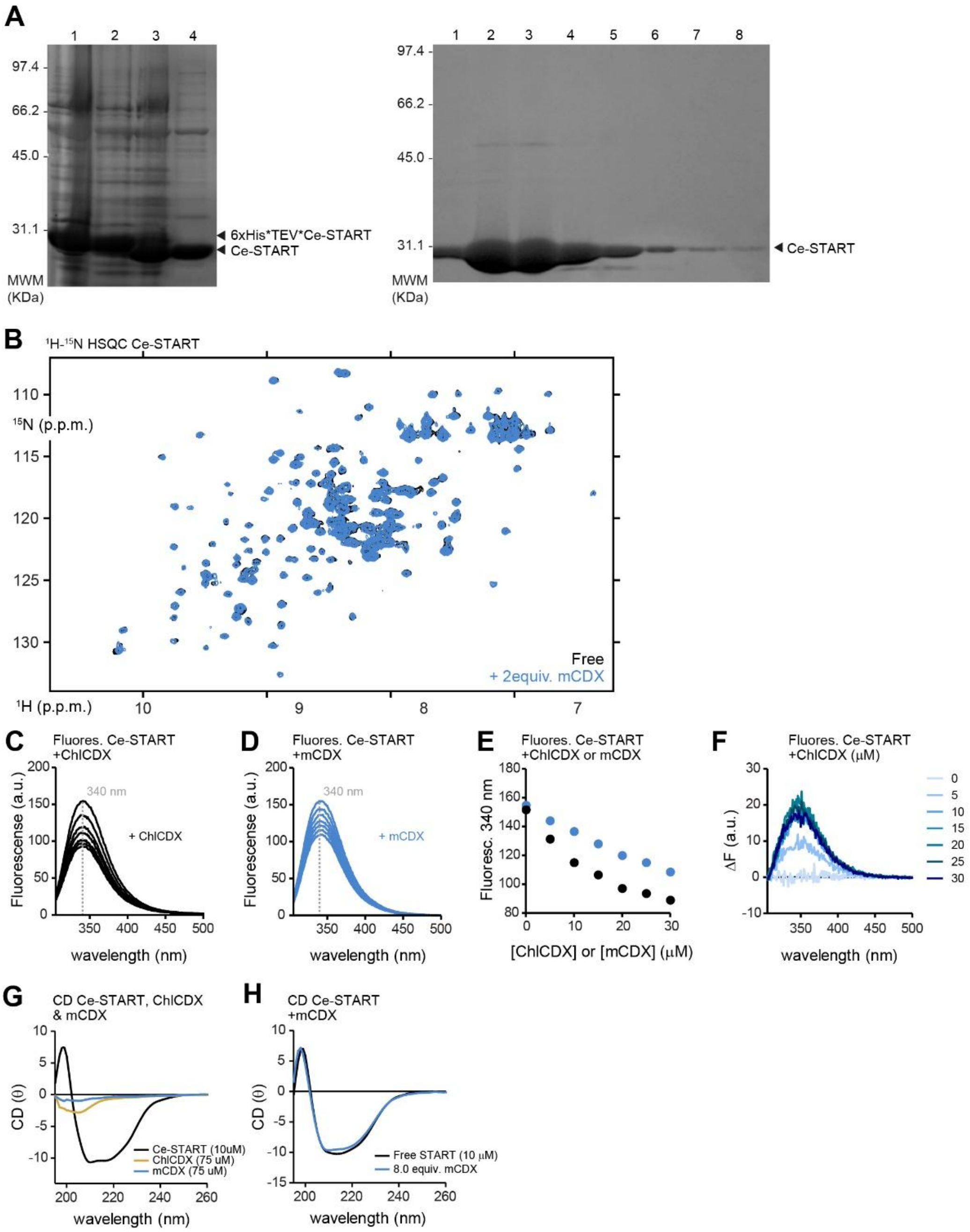

Supplementary Figure 4. (A) SDS-PAGE analysis of the purification of recombinant Ce-START. Left panel shows the removal of the 6xHis-TEV tag. Lane 1, corresponds to 6xHis-TEV-Ce-START; lane 2 6xHis-TEV-Ce-START + TEV protease before cleavage; lane 3 6xHis-TEV-Ce-START + TEV protease after cleavage and lane 4 Ce-START purified from the 6xHis-TEV tag using Ni-NTA resin. Right panel shows, the SEC elution profile of Ce-START. Fractions, 2-5 were pooled and concentrated for further analysis. The mature protein contains a Gly at position 1 from the TEV processing site. **(B)** Overlay of ^{1}H-^{15}N HSQC spectra of 150 μM, ^{15}N-isotopically

enriched Ce-START in the absence (black) and presence (cyan) of 2 equivalents of cholesterol-free methyl-cyclodextrin (mCDX). This corresponds to the same amount of mCDX complex to cholesterol **(C, D)** Intrinsic Trp fluorescence spectra of 10 μM Ce-START in presence of cholesterol-mCDX **(C)** or mCDX **(D)**. **(E)** Intrinsic Trp fluorescence of 10 μM Ce-START at increasing concentrations of ChlDX and mCDX. Fluorescence intensity maxima at 340 nm were obtained from the experiments showed in panels **(C, D). (F)** Difference fluorescence spectra of Ce-START-Chl complex after subtraction of the mCDX contribution from each added concentration. **(G)** Far-UV CD spectra of 8 μM Ce-START and of 75 μM of Chl:CDX (yellow) and 75 mM μCDX (cyan). **(H)** Far-UV CD spectra of 8 μM Ce-START in the absence (black line) or presence of 8 equivalents of mCDX (cyan).

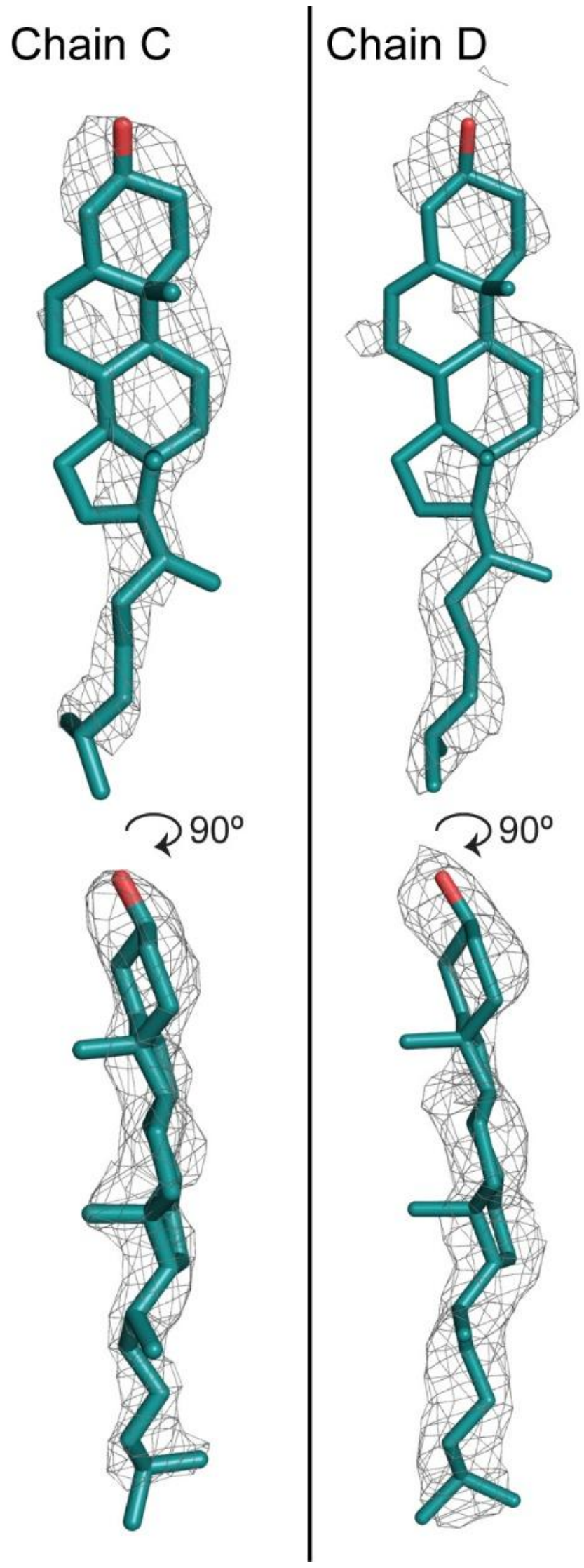

Supplementary Figure 5. *2mFo-DFc* electron density for the cholesterol molecules bound to Ce-START (Ce-START:Chl; PDB 13FR). The cholesterol molecules modelled in chain C (left) and chain D (right) are shown in stick representation, with carbon atoms in dark cyan and oxygen atoms in red. The corresponding *2mFo-DFc* electron density map, contoured at 1 σ, is displayed as a gray mesh around each ligand. Top panels show the molecules viewed along the plane of the steroid ring system; bottom panels show the same molecules rotated 90° around the long axis of the steroid. The asymmetric unit contains four protein chains (A-D); residual electron density compatible with a cholesterol molecule was observed only in chains C and D, and is shown here in both orientations to support ligand placement at these two sites. The mean B-factor of the cholesterol molecules modelled in chains C and D is 64.2 Å² and 64.1 Å², respectively, compared with a mean B-factor of 41.42 Å² for the protein as a whole. The resulting B-factor ratio

B(ligand)/B(protein) of ~1.55 lies within the range typically associated with bound ligands displaying moderate residual mobility, supporting the assignment of cholesterol at these two sites despite the discontinuous appearance of the *2mFo-DFc* density in some regions of the ligand.

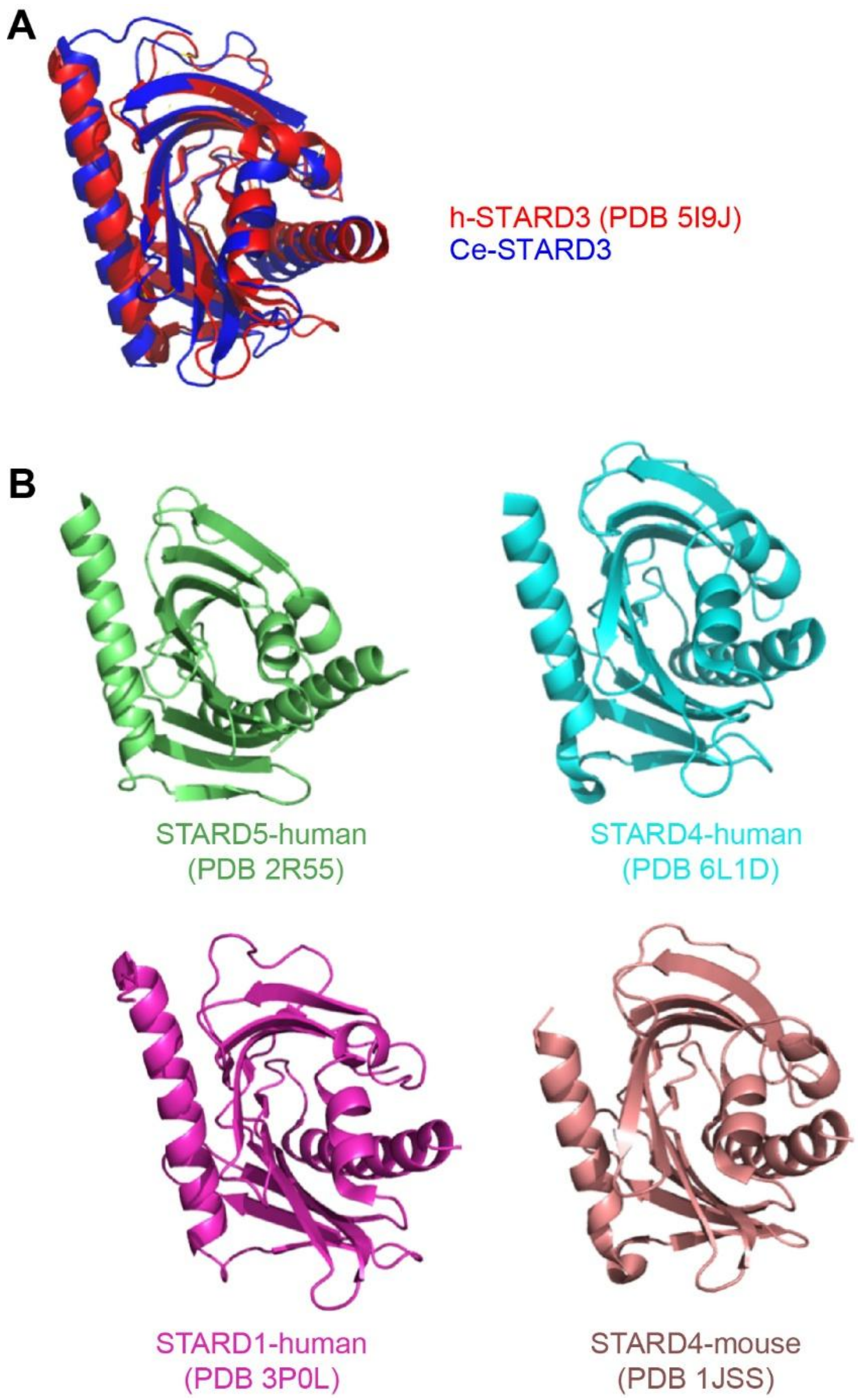

Supplementary Figure 6. (**A**) Overlay of X-ray crystal structures of human (red) and *C. elegans* (blue) START domains. Calculated RMSD was 1.3 Å over 103 atoms. (**B**) X-ray crystal structures of START domains from different STARD proteins.

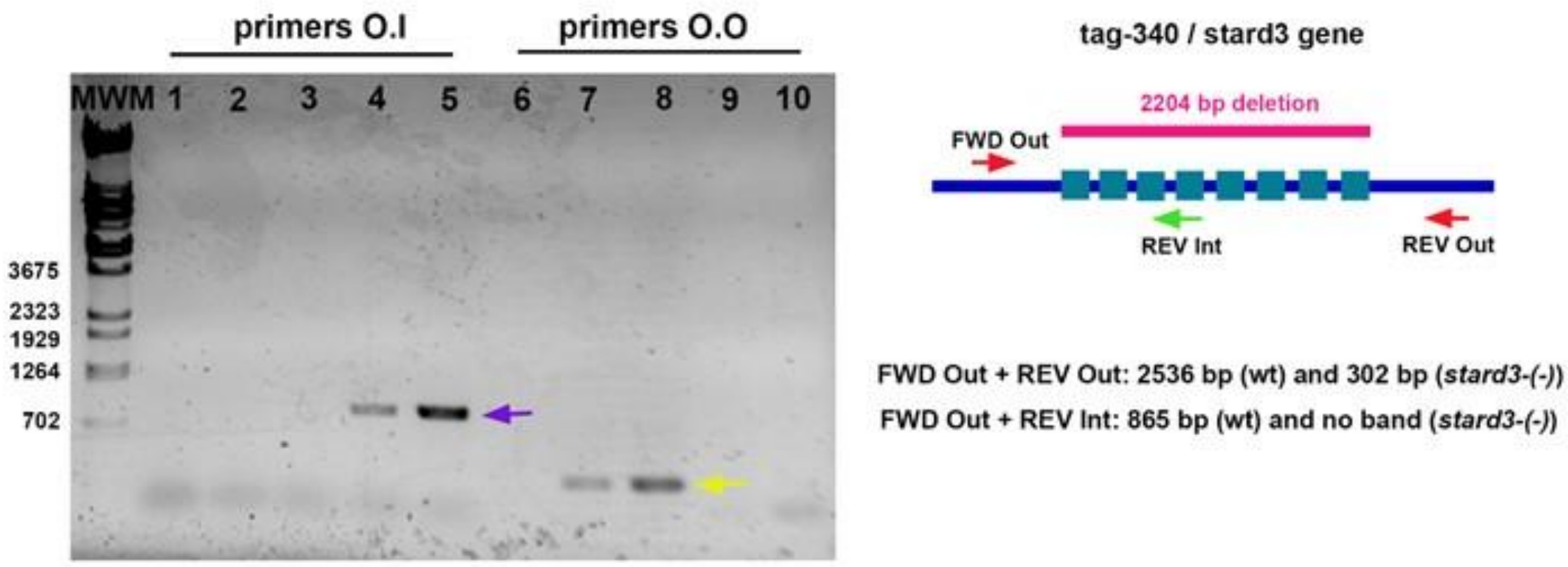

Supplementary Figure 7: (A) Agarose gel showing PCR amplification of the *stard3* sequence in wild type (N2) and the *stard3-(-)* mutant. Lanes 1 and 6: no DNA (negative control); 2 and 7: *stard3-(-)* DNA, 1/100 dilution; 3 and 8: *stard3-(-) DNA*, 1/10 dilution; 4 and 9: wild-type DNA, 1/100 dilution; 5 and 10: wild-type DNA, 1/10 dilution. Primers O.I: FWD Out + REV Int; primers O.O: FWD Out + REV Out. Molecular weight is expressed in base pairs. The purple and yellow arrows indicate the 865 and 302 bp bands, respectively. It was not possible to detect the 2536 bp band that should be amplified from Primers FWD Out and FWD Int in the wild type genotype due to the low efficiency of the standard PCR reaction. **(B)** Schematic representation of the *tag-340/stard3* gene in *C. elegans*. Light blue boxes represent exons. The genetic deletion in *stard3-(-)* is represented in magenta and the primers used for PCR confirmation are depicted as red and green arrows.

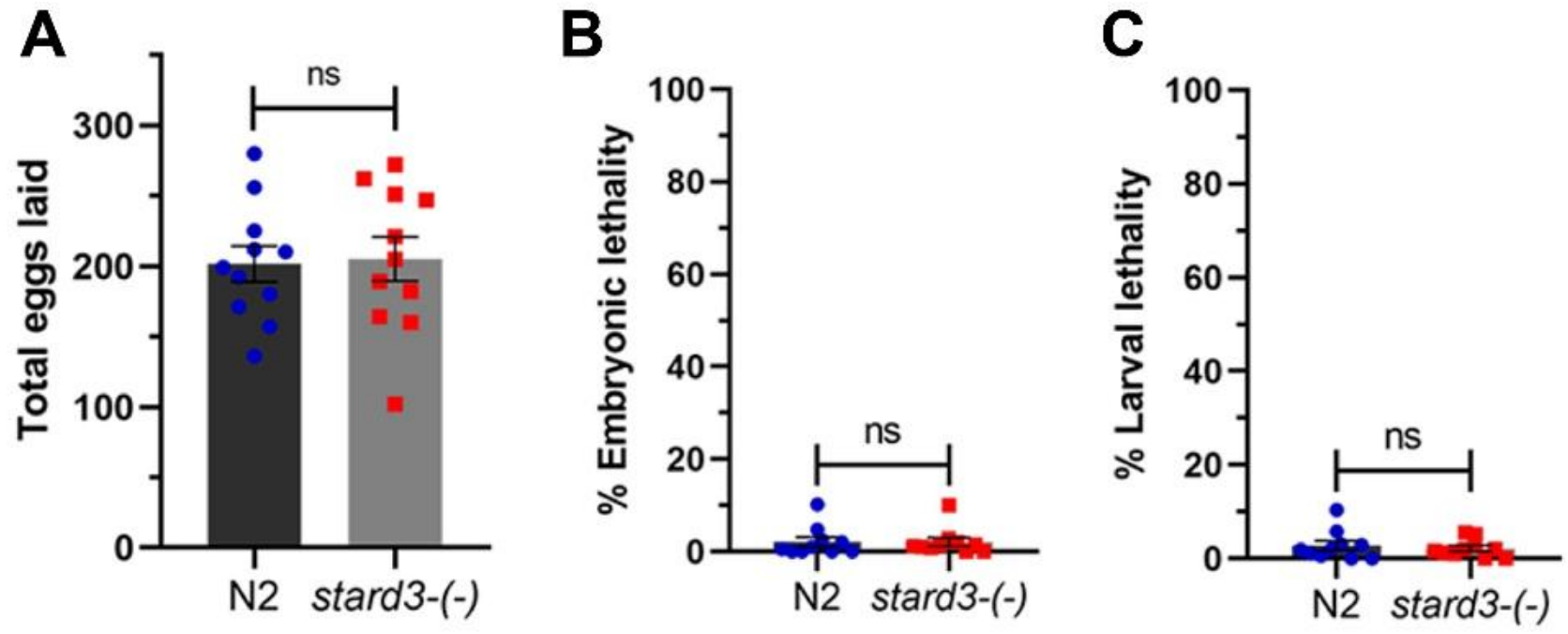

Supplementary Figure 8. Mean number of eggs laid **(A)**, percentage of embryonic lethality **(B)**, and larval lethality **(C)** are shown for N2 and *stard3-(-)* worms grown on standard NGM medium (supplemented with 13 µM of cholesterol) at 20ºC. A minimum of 10 P0 worms were analyzed for each condition. Error bars represent SEM.
